# Waste not, want not: Value chain stakeholder attitudes to surplus dairy calf management in Australia

**DOI:** 10.1017/awf.2024.4

**Published:** 2024-02-22

**Authors:** Sarah E Bolton, Bianca Vandresen, Marina AG von Keyserlingk

**Affiliations:** 1 Animal Welfare Program, Faculty of Land and Food Systems, The University of British Columbia, 2357 Main Mall, Vancouver, BC, V6T 1Z6, Canada; 2 Melbourne Veterinary School, Faculty of Science, The University of Melbourne, Parkville, VIC, Australia

**Keywords:** animal welfare, beef, bobby calves, dairy cattle, perspectives, supply chain

## Abstract

The management of surplus dairy calves in Australia has traditionally been influenced by the economic viability of different practices. When beef prices are favourable, more surplus calves are raised for beef, and when beef prices are low, more calves are killed in the first few days of life. Early life killing of surplus calves may however threaten the dairy industry’s social licence to operate. The aim of this study was to describe the views of value chain stakeholders regarding the management of surplus calves. Representatives from seven post-farm gate organisations participated in semi-structured interviews and were asked about their views on current practices, alternatives to early life killing and how best to implement change. Responses were analysed using inductive thematic analysis and were organised into three themes: (1) ethics of surplus calf management; (2) economics of surplus calf management; and (3) moving towards solutions including approaches to affecting change. We conclude that stakeholders widely recognised early life killing of surplus calves as a threat to the industry’s social licence. Whilst technical solutions such as beef on dairy breeding programmes were cited as important, participants emphasised that implementing sustainable solutions will require collaboration, leadership, and commitment by all stakeholders along the value chain.

## Introduction

**Figure 1.**
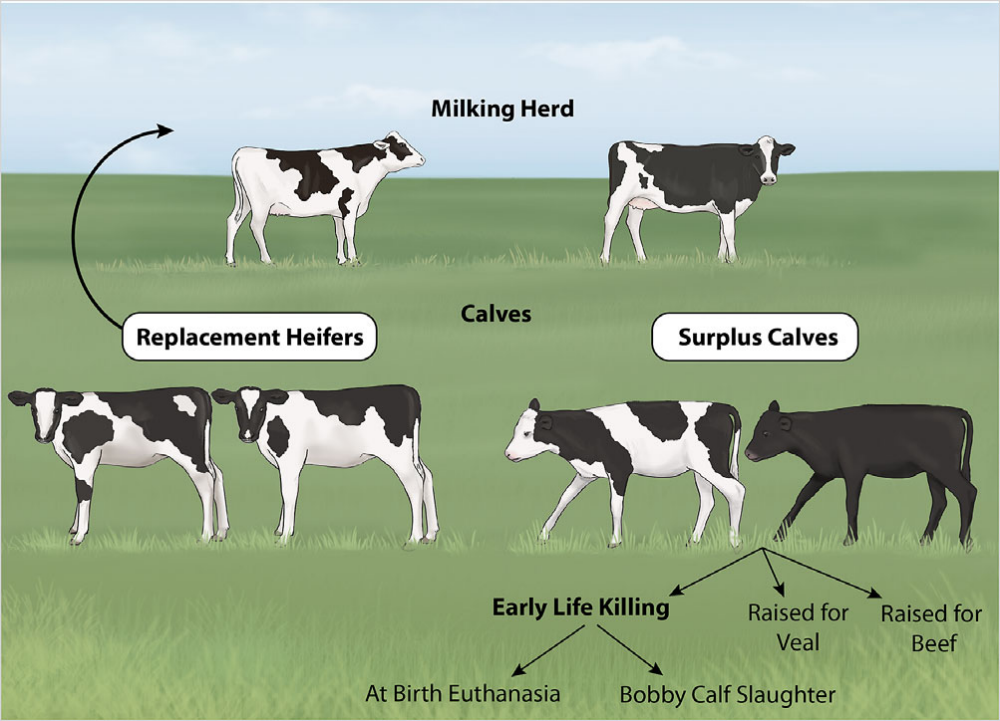
Management pathways for dairy calves. Dairy cows usually give birth to a calf once a year. The calves are generally separated immediately after birth. Some female calves are used as future replacement heifers and will later join the milking herd. Male and surplus female calves may be raised for beef or veal. Alternatively, they may be managed through early life killing; either being euthanased on-farm at birth, or transported for slaughter as ‘bobby calves’ within the first few days of life. (Illustration by Ann Sanderson, independent illustrator, Canada).

The primary business focus of most dairy farms is producing milk, with fluctuations in milk price usually being the greatest factor impacting farm revenue (Wolf *et al.* 2009). To ensure high levels of milk production, most dairy cows are managed such that they give birth annually, initiating the onset of a fresh lactation. Although many female calves remain on the farm as replacements for the lactating herd, some female and all male calves born are surplus to herd replacement requirements. Depending on the region and operating environment, these ‘surplus’ (sometimes termed ‘non-replacement’) dairy calves are managed through different pathways including being raised for veal or dairy beef, or through early life killing. In Australia, early life killing includes at-birth euthanasia of healthy calves on-farm (Haskell 2020), followed by disposal, as well as calves that are transported to slaughter as 5–30 day old ‘bobby calves’, a term used in legislation to describe calves under 30 days of age that are transported without their mother (Animal Health Australia 2012) (Figure 1).

Given that many surplus calves and most cows culled from the dairy herd are eventually slaughtered for meat, dairy farmers are also contributors to the beef value chain (von Keyserlingk *et al.* 2013); ‘value chain’ defined as *“the full life cycle of a product or process, including material sourcing, production, consumption and disposal”* (World Business Council for Sustainable Development [WBCSD] 2011; p 3). The contribution of beef from the dairy herd (either from cull cows or surplus calves slaughtered for meat) to a country’s overall beef production varies regionally. In Australia and Canada about 10 and 22% of the beef produced, respectively, originates from dairy farms (CANFAX 2022; Meat and Livestock Australia [MLA] 2022). This is much higher in the European Union (EU), given that about 65% of cattle in the EU are dairy breed animals, the majority of which will eventually contribute to the beef supply (Greenwood 2021; Vinci 2022).

Whether surplus calves are destined for early life killing or enter the beef value chain as veal or dairy beef is dependent in large part upon the type of dairy production system and demand for beef. Historically, predominantly pasture-based, seasonal calving dairy regions that are net exporters of beef, such as Australia and New Zealand, have been more likely to employ early life killing of surplus calves (Boulton *et al.* 2020; Dairy Australia 2023b). In contrast, regions that rely more on year-round calving dairy systems and have stronger demand for domestic beef production, such as North America and Europe, are less likely to employ early life killing of surplus calves (Boyle & Mee 2021). In Australia, annual fluctuations in the numbers of calves killed in the first few days of life are influenced by commodity prices. When beef prices are low, more calves are slaughtered in the first week of life (as high as > 500,000 head in the 2014/2015 fiscal year) (Dairy Australia 2023b), demonstrating that calves are managed according to the path of least economic risk. However, even in times of relatively high beef prices, such as the 2021/2022 fiscal year, over 200,000 calves were slaughtered at 5–30 days of age (Dairy Australia 2023b), with an additional 5% of calves self-reported by farmers as being euthanased at birth, on-farm (Dairy Australia 2023a). Calves destined for early life slaughter are vulnerable to compromised welfare on farms, during transport and at abattoirs. Stressors include food and water deprivation, disease, injury, stress from handling, social mixing, and new environments (for a review, see Creutzinger *et al.* 2021; Roadknight *et al.* 2021). Moreover, the practice of early life killing of a healthy calf presents an ethical dilemma that is separate to scientific animal welfare implications (Haskell 2020).

Early life killing of surplus calves has been argued by some to be out of step with public values (Ritter *et al.* 2022), bringing into question the industry’s ability to retain its social licence to operate (Bolton & von Keyserlingk 2021). Originating out of the mining industry, the concept of a social licence was introduced to encompass the non-regulatory political and social risks presented to an industry or business outside of any formal licencing or permits required (Cooney 2017). The loss of a ‘social licence’ refers to the withdrawal of societal support for an industry or practice, often resulting from outrage and associated loss of trust when the broader community learns of a practice or event that is out of step with their expectations (Hampton *et al.* 2020).

Livestock production systems are fraught with issues that many stakeholders find contentious (Weary *et al.* 2016), giving rise to moral dilemmas; situations that arise when societal values conflict with livestock production practices, often resulting from unintended consequences (Gremmen 2020). Some of the most challenging moral dilemmas in animal agriculture include the killing of day-old chicks (de Haas *et al.* 2021) and early life killing of surplus calves (Haskell 2020). Routine highly contentious agricultural practices that are entrenched within the system have been referred to as a type of moral “lock in” (Bruijnis *et al.* 2015). These “locked in” practices are difficult and costly to change, often involve choosing the least of several possible evils (Gremmen 2020), and in some specific cases are argued to meet the criteria of “wicked problems” (Bolton & von Keyserlingk 2021). The challenge for the global dairy industry going forward is to implement sustainable management strategies for surplus calves that are both economically viable and socially acceptable. In practice, this means prioritising pathways for surplus calves that provide them a reasonable length, quality, and purpose of life (Ritter *et al.* 2022).

The implementation of economically viable alternatives to early life killing of surplus calves, such as raising them for beef, is a persistent challenge. For example, over 200,000 bobby calves were slaughtered at 5–30 days of age in the 2021–2022 fiscal year despite record high beef prices (Dairy Australia 2023b), and continued growth in the sales of sexed semen and beef semen to dairy farms employing beef on dairy breeding strategies (National Herd Improvement Association of Australia 2022). Implementing sustainable beef pathways for surplus dairy calves is made even more challenging by a lack of clarity regarding the needs of multiple beef and dairy value chain stakeholders. These stakeholders include, but are not limited to, genetics companies, calf growers/rearers, beef finishers, dairy processors, beef packers and retailers. Given the diversity of stakeholders, the complexity of the value chain and little knowledge of their different perspectives, it is not surprising that technical solutions attempting to solve this issue (e.g. sexed semen and beef on dairy breeding strategies) have failed to create widespread, sustained change. Moreover, as Bolton and von Keyserlingk (2021) argue, issues that meet the criteria for a wicked problem require transformational change. In essence, the problem of early life killing of surplus calves must become an “unlocked” moral issue (Bruijnis *et al.* 2015), as only then will sustainable solutions be identified and implemented.

Many different approaches have been suggested for tackling wicked problems. One element that most approaches have in common involves identifying and working with the needs of all stakeholders (Bolton & von Keyserlingk 2021). As such, the aim of this study was to describe the views of post-farm gate beef and dairy value chain stakeholders regarding surplus calf management practices in Australia and how the industry might implement alternatives that are socially acceptable and economically viable into the future.

## Materials and methods

### Ethical approval

This study was approved by The University of British Columbia (UBC) Behavioral Research Ethics Board (protocol no H18-02880-A012) and the University of Melbourne Human Research Ethics Board (protocol no 20750). All participants provided written consent to participate.

### Positionality statement

In qualitative research, the researchers’ experiences can also influence the research process, as researchers act as instruments for collecting qualitative data (Holmes 2020). SB is a female PhD student at The University of Melbourne and a visiting scholar with the UBC Animal Welfare Program (AWP). She grew up on beef and dairy farms in Australia before obtaining her Bachelor of Veterinary Science degree. She has since worked in veterinary practice and has experience in dairy farm management and calf rearing. At the time of data collection for the present study, she was the National Animal Welfare Lead at Dairy Australia. BV is a female PhD student at UBC AWP. She was born and raised in Brazil, where she obtained her Bachelor of Science degree in veterinary medicine and her MSc degree in Animal Welfare. She did not grow up in a farming community but has lived experience working at The UBC Dairy Education and Research Centre. MvK is a female Professor at UBC where she has co-led the UBC AWP since 2002. She grew up on a beef cattle ranch in Canada and worked in the agribusiness sector for seven years before joining the UBC AWP as a Professor in 2002.

### Participant recruitment

Participants selected for this study were individuals who worked in management roles for organisations known to play a key role in either the Australian beef and/or dairy value chains. Participants were initially identified via email or phone through existing networks held by SB. Those that agreed to participate were informed of the study details and asked to sign the consent form (via email). Initially, ten companies were contacted but two stated that they were not interested in participating and one elected to participate but did not sign the consent form (and was thus not included in the study). The seven organisations that participated comprised one genetics company, one beef finisher, two beef packers, two milk processors and one retailer, forming a convenience sample.

The first version of the semi-structured interview guide was prepared by SB and revised by MvK. The interview guide was then circulated for comment to three individuals, all of whom had previous experience with undertaking interviews but were not subject experts. All comments received were discussed and the interview guide revised accordingly (see Supplementary material).

In total, 12 representatives from seven organisations (ranging from 1–4 participants each) were interviewed via Zoom (Zoom Video Communications Inc 2021) by SB in partnership with an external consultant experienced in market research interviews. Each interview lasted between 60 and 90 min. After initial introductions, the participants were asked for consent for the meeting to be recorded. The interview started by asking the participants about their understanding of the surplus calf issue and whether their company had ever held discussions on how to address it. This was followed by a brief overview of a wider participatory framework project exploring the surplus calf challenge involving farmers, pre-farm gate advisors and community members. Interview participants were informed that the results of their interviews would be de-identified and shared with the participants of the participatory framework project and prepared for publication.

To elicit further discussion, participants were asked to provide feedback on four possible future scenarios involving different approaches to addressing the surplus calf issue in Australia (Table 1). The scenarios were created to reflect a broad range of different high-level approaches, based on common themes in industry-level discussions held by SB during the three years she worked for Dairy Australia. Participants were then asked about their views on responsibility, co-operation, and collaboration along the value chain to address the surplus dairy calf issue. Lastly, participants were asked whether they would like to provide any additional input at the end of the interview.

**Table 1.** Descriptions of four broad-ranging hypothetical future scenarios involving different approaches to addressing the surplus calf issue in Australia used to elicit discussion with study participants on approaches to sustainable surplus calf management

Scenario description
All surplus calves are grown to adulthood for the beef supply chain. This is able to be done because the costs are covered either through milk price increase or supply chain co-operation.
Hormones are introduced to enable cows to continue to produce milk without having to repeatedly become pregnant, meaning surplus calves are never born in first place.
The local dairy industry transforms into a connected network of farms that work together on a solution. The solution differentiates the region’s milk and/or dairy beef and attracts a premium.
Farmers use technology that is currently available including sexed semen to reduce male calves being born. The remaining calves either enter the dairy industry or are grown to adulthood with optimised nutrition and production for the beef supply chain.

### Data analysis

Audio recordings of seven interviews were transcribed by a professional transcription service (Rev.com Inc, Austin, TX, USA). All identifying information was removed from the transcribed data, including the names of participants, the organisations they represented and any quotes that made themselves or their organisations identifiable. Where there was more than one participant in an interview, efforts were made to separate the quotes by individual participants. The transcriptions were identified by the interview number only (i.e. Interview 1).

All transcribed data were submitted to thematic analysis using an inductive approach (Braun & Clarke 2006). Inductive thematic analysis is based upon the creation of codes without a pre-existing coding frame, where the collected data drives the analysis, rather than the analysis being driven by pre-existing theory or questions defined in the interview guide, as is used for deductive analysis (Clarke & Braun 2017). Inductive analysis was selected to understand how participants viewed the broad-ranging complexities of surplus calf issue (Bolton & von Keyserlingk 2021). That value chain stakeholder attitudes to this issue have been relatively under-studied was further reason to select inductive analysis over deductive.

The development of the codes began with the extensive reading of the transcripts and familiarisation with the data until patterns were identified. Both SB and BV independently developed the initial codes, then discussed their findings. During this process, the codes were reviewed and refined throughout the data analysis (i.e. specific codes were merged into broader codes and *vice versa* until clusters of similar codes were developed). The code clusters were merged into sub-themes, which were connected into broader themes. Intercoder reliability (between BV and SB) was established, wherein both authors coded the data independently using the draft codebook followed by discussions where they compared results and refined the codes and themes. The codebook was further reviewed through in-depth discussions with all authors to address any remaining discrepancies, before the final codebook was agreed upon (see Supplementary material). BV then used the final codebook to code all the transcripts using NVivo (version 12, QSR International Pty Ltd, USA). In the following section we describe each of the themes and provide quotes, some of which were modified for clarity, to exemplify ideas from each of the sub-themes.

## Results and Discussion

**Figure 2.**
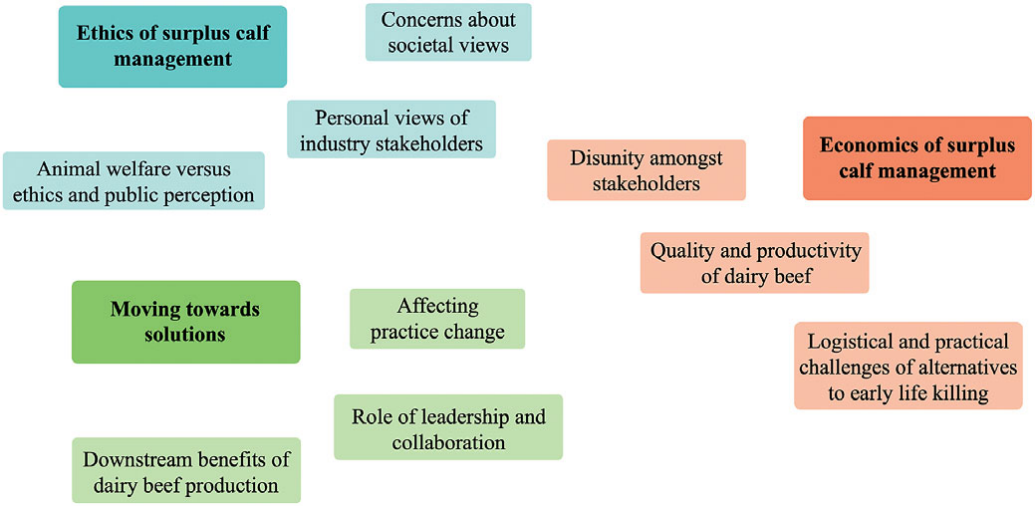
Thematic map of themes and subthemes from interviews with 12 representatives from seven organisations from the Australian beef and dairy value chain on their attitudes towards surplus dairy calf management. Boxes in blue represent codes within the broader theme of ethics of surplus calf management. Boxes in orange represent codes within the broader theme of economics of surplus calf management. Boxes in green represent codes within the broader theme of moving towards solutions.

Participants discussed three main themes regarding the management of surplus calves in Australia, each with three underlying sub-themes (Figure 2). The themes were: (1) Ethics of surplus calf management, including concerns about societal views, personal views of industry stakeholders, and the animal welfare, ethical and public perception implications of different practices; (2) Economics of surplus calf management, including disunity amongst beef and dairy industry stakeholders, opportunities to improve the quality and profitability of dairy beef, and logistical and practical challenges of alternatives to early life killing; and (3) Moving towards solutions, including affecting practice change, the role of leadership and collaboration, and downstream benefits of dairy beef production.

### Ethics of surplus calf management

Participants frequently defined early life killing of surplus dairy calves as an ethical issue with societal views being their primary concern, but also provided their personal ethical concerns regarding the issue. Participants also explored the complex relationship between animal welfare and ethics regarding the practice of early life killing.

#### Concerns about societal views

Many participants believed that early life killing of surplus dairy calves poses a risk to the dairy industry’s social licence. In the words of one individual *“…* [the bobby calf issue] *cuts to the heart of the social licence to continue to dairy”* [Interview 2]. In terms of a business risk, one participant was concerned that *“if there was poor welfare practice, such as treatment of bobby calves in some way it would definitely be a risk to us”* [Interview 5]. Some organisations also conveyed that: “*We no longer do that* [slaughter bobby calves] *as* [it is] *a risk to our business and from our customers’ perspective […] and there was certainly pressure from the companies that we deal with for us to drop the bobby calf kill*” [Interview 4].

The concerns expressed by the participants regarding risk are not unfounded, given that once aware, the public will likely not support early life killing of calves. In an online survey, only 3% (of 998 participants) stated that they regarded slaughtering calves less than one month of age as appropriate (Ritter *et al.* 2022). Whilst some countries have already worked towards halting the practice of at-birth euthanasia of healthy calves on-farm (e.g. Great Britain; [Agriculture and Horticulture Development Board; AHDB 2020]), to our knowledge, no country to-date has banned early life slaughter of surplus dairy calves for meat.

Participants commented on the contribution of social media and undercover exposés to the vulnerability of the dairy industry. In the voice of one participant “*I think the consumer is more aware that you can’t simply tell a story about the management of female calves anymore and I think the rise of social media again makes everyone clear what the vulnerability is*” [Interview 2], particularly, if “*someone happens to get pictures [‥] and it hits the front page”* [Interview 7]. The fear of undercover media exposés is not surprising nor unwarranted. Exposés have increased the discussion of farm animal welfare in other regions (United States; Shields *et al.* 2017) and in Australia (e.g. the ban on live cattle export to Indonesia; Schoenmaker & Alexander 2012). Interestingly, some participants acknowledged the importance of working to avoid this risk altogether by, *“make*[ing] *sure that we’re ahead of the game or otherwise* [it’s] *just another reason for a bad news story to hit mainstream media”* [Interview 1].

Concerted efforts to implement minimum standards for some aspects of animal care have been made, such as prohibiting the use of blunt force trauma as a method of euthanasia (e.g. Australia [Animal Health Australia 2016; Australian Dairy Farmers 2020]; New Zealand [New Zealand Ministry of Primary Industries 2016]; Canada [DFC-NFACC 2023]). However, compliance with these types of minimum standards will likely not be sufficient to maintain public trust if the practice fails to resonate with societal values (Weary & von Keyserlingk 2017). For instance, a New Zealand milk processor recently announced that effective from June 30, 2023, they would no longer purchase milk from farms that euthanase healthy calves on-farm (Fonterra 2022).

Participants also mentioned the possible risk of compromising the good image of the Australian agriculture: *“Really as a country…we are seen to be really good on the animal welfare status and we are seen as that clean green* [image] *and that’s not just from the dairy industry, that’s from the beef industry, that’s Australia’s agricultural industry as a whole”* [Interview 1]. The use of pastoral images to promote dairy farming have long been used by the Australian dairy industry (Phillipov & Loyer 2019), where the majority of a cow’s diet comes from grazed pastures (Dairy Australia 2023c). Promotion of the pastoral image by the agricultural industry has been linked to the long-standing support given to this sector by its citizens (Cockfield & Botterill 2012).

Potential links between surplus calf management practices and cow-calf separation (for a description, see Meagher *et al.* 2019) were also raised by our participants. In the words of one participant: “*I think if you’re a* [member of the] *general public and you see these baby cows being transported, lots of people will see them, baby calves without their mums being taken on this scary journey*” [Interview 7]. Although awareness of cow-calf separation is generally low amongst the public (Ventura *et al.* 2016), once aware, there is little to no support for this practice (i.e. Hötzel *et al.* 2017; Sirovica *et al.* 2022). Although the public generally exhibit negative attitudes towards both practices, attitudes towards early life killing have been shown to be more negative than cow-calf separation (Ritter *et al.* 2022).

#### Personal views of industry stakeholders

That individual participants grappled with the ethics of early life killing was evident as they conveyed their own personal views and experiences. One participant, who entered the dairy industry in adulthood, described being shocked when learning about early life killing of surplus calves:

*“I’d drank milk my whole life and I never actually even considered* [where] *it comes from, except for all those children’s books you read where it’s all about a happy mum cow making the milk […]. It really was a shock to the system when I entered the food industry and realised where my food came from and I thought that I was educated coming into it that, having gone to university and studied the food industry and all these things, that it never even actually came up in my curriculum while I was studying” [Interview 7].*

Another participant who had grown up in the industry stated that “*I increasingly can’t come to terms with the early age euthanasia of calves just because they’re not going to be reared. That’s something that we’ve put in place and we’re saying it’s okay, […] well I personally just keep reconsidering that and I just don’t find it acceptable in the long term*” [Interview 5].

Participants also acknowledged that early life killing of calves was a matter of concern for farmers. However, despite the practice being commonplace, participants believed that farmers found the task undesirable: *“I have never seen anyone enjoy euthanasing an animal […], people want to do the right thing.*” Indeed, as another participant said, *“I think a lot of farmers […] think that what they are doing is best practice and I do not think anybody intends to do the wrong thing or something that is not humane or economical or socially acceptable”* [Interview 7]. Others have highlighted cognitive dissonance as the reason explaining the disconnect between farmer beliefs or values and what they do in practice (Neave *et al.* 2022).

The role of farmers’ mental health was also acknowledged: *“The mental health and well-being of a farmer is fundamental to the level of assurance you’re going to get about how they manage the well-being of their animals”* [Interview 2]. Looking at the Australian dairy industry, 92% of dairy farms are family owned with only 45% of those working in the business reporting the dairy industry provides them an effective work-life balance (Dairy Australia 2021). A lack of work-life balance and associated fatigue for people working in and on the business may contribute to reduced mental health which has been linked to poor animal welfare outcomes (King *et al.* 2021), underpinning this as a key area of focus for addressing the long-term sustainability of the industry (Kato *et al.* 2022).

Social sustainability is often discussed in the context of society’s views about farming (von Keyserlingk *et al.* 2013). However, our results emphasise that industry stakeholders also have their own ethical concerns about early life killing of calves, forming an additional dimension that should not be overlooked (Segerkvist *et al.* 2020).

#### Animal welfare versus ethics and public perception

When managing surplus calves through early life killing, participants voiced that the interplay between animal welfare impacts and ethical views of different stakeholders was complex. “*Some people may have the view that actually humanely euthanasing on-farm is actually kinder to the calf if you can’t take it onto somewhere else* [while] *other people may say actually we’re completely against euthanasing*” [Interview 7]. Whilst some argued that correctly performed at-birth euthanasia avoided welfare challenges, they acknowledged the ethical dilemma this stance created.

*“If euthanasia is being conducted in* [an] *appropriate fashion by an appropriately trained staff member and […] blunt force trauma is avoided except when there’s absolutely no other option* [then welfare compromise can be avoided]. *But […] if someone did a dump* [launched an exposé] *on us it’s going to look really bad and murdering baby calves – that can never have a good look even when it’s done humanely”* [Interview 5].

At-birth euthanasia was also seen by some to offer a better welfare outcome in some situations. As one participant said “*I think also […] what’s really important is […] ensuring that these calves are getting the correct welfare. There’s no point in rearing a calf if it’s going to have terrible welfare*” [Interview 7]. Indeed, whilst some argue that when an animal is euthanased correctly there is no compromise to its welfare (Walker *et al.* 2020), the context under which the animal is killed may still be at odds with a person’s core values (Ritter *et al.* 2022).

Conversely, some participants saw calves slaughtered in the first few days of life and directed into the food chain as justified because the animal is not wasted. As stated by one participant: “*I think it’s easier to make the concept palatable if there is a purpose in the death so a calf going to become a foodstuff is an easier sell than a calf that just goes into a hole in the ground*” [Interview 5]. This belief led some participants to argue that the challenge lies in educating the public into understanding this concept, for instance, one participated stated:

*“In certain areas I think we’ve actually got it right if we’re going to continue with the belief that an appropriate welfare-friendly, sustainable practice is early destruction of male calves to go into the meat industry and the industry seems comfortable with that. It’s whether we can perpetrate that belief into the wider community”* [Interview 5].

Previous attempts to educate the public into accepting ‘contentious routine dairy practices’ have failed given that the practices themselves were at odds with public values (Ventura *et al.* 2016; Hötzel *et al.* 2017). In contrast, one individual argued that: *“[…] early slaughter is not a good message…, it is still a baby calf being killed for meat production which, I […] think is really hard one to try and communicate”* [Interview 7]. Many participants acknowledged that public perception is a key determinant of sustainable surplus calf management practices.

“*I think in terms of the bad news story,* [the question is] *would it pass the pub* [public approval] *test? I think if the bobby calves have been grown out* [for beef] *and there’s a story behind it* …[and] *it’s not that they’re being euthanased on-farm or just sent straight to slaughter then I think it’s a nice story*” [Interview 7].

Our findings indicate that early life killing of young dairy calves is an ethical dilemma for many industry stakeholders, with some justifying early life slaughter of surplus calves while others felt there was no justification for early life killing.

### Economics of surplus calf management

Participants frequently emphasised that economic viability is at the heart of the surplus calf challenge and that it is both a key driver of the *status quo* and a barrier to sustainable change. The traditional separatism of the Australian beef and dairy industries was highlighted as a key contributor, along with the logistical and practical challenges of implementing alternatives to early life killing. Participants also highlighted several challenges and opportunities to improve the quality and productivity of dairy beef as a viable alternative to early life killing.

#### Disunity amongst stakeholders

Participants acknowledged that “*most* [dairy] *farmers don’t see past the farm gate”* [Interview 7] and this limited understanding of beef supply chains impacted their ability to implement economically viable dairy beef systems. Participants often attributed this to a lack of *“[…] tools or the ability* [of farmers] *to follow* [dairy beef] *programmes for these sort of cull animals”* [Interview 3]. Participants also highlighted that *“Typically* [it’s been assumed that] *a dairy cross or a dairy steer […] won’t get the weight gain* […and this] *will affect your production”* [Interview 1]. This, in turn, was seen to perpetuate negative attitudes towards dairy beef. For example, one participant stated that “*As soon as you say dairy cross, most feedlots and abattoirs just switch off straight away”* [Interview 6].

The perceived lack of unity described by our participants between the beef and dairy sectors has been attributed by some to dairy farms traditionally viewing their businesses as one of producing only milk and thus in isolation of being able to contribute to beef production (Wolf *et al.* 2009). Some called for greater emphasis on the different contributions that beef production could add to the dairy business *“[…] this calf […] is an offshoot which should add value”* [Interview 3]. However, some argued that for dairy beef to be viable there needed to be *“[…] some* [economic] *margin*” [Interview 1].

Participants believed there to be opportunity for the dairy and beef industries to co-operate in directing surplus calves into beef production, creating mutual benefits. However, it was also emphasised that “*[…] a lot of people just don’t understand that we need to utilise what the dairy industry has got […]* [and] *we need to utilise it efficiently*” [Interview 3]. Participants believed that improved understanding by the farmers on the needs of the value chain was necessary to make this co-operation possible. However, they also conveyed that everyone involved, including the farmer, must accept *“their responsibility in the supply chain*” [Interview 1]. The role of the farmer was viewed as key:

*“*[Dairy farmers] *need to understand what that target market is and what they can do within their breeding operation to ensure that they’ve got* [a calf] *that’s feasible for someone that [a] they’re not going to get docked* [discounted] *because it’s a dairy steer, or [b] because they know it’s going to hit the economical pocket that they’re doing something to progress and move forward in that space to ensure they’ve got a pathway*” [Interview 1].

Calls for increased collaboration between the cattle industries and the associated beef value chain has been raised by others (AHDB 2020); embedded within this collaboration is the need to ensure that any negative cultural attitudes amongst beef stakeholders toward surplus dairy calves as beef animals are overcome.

#### Quality and productivity of dairy beef

Participants identified challenges and opportunities in maximising the quality and productivity of dairy beef production with an emphasis on breeding and managing calves according to market demands. In selecting beef genetics to maximise the viability of surplus calves for beef production, participants noted that whilst dairy breeds often exhibit valued meat quality traits such as marbling (Pfuhl *et al.* 2007), they can perform poorly in terms of carcase yield and conformation (Clarke *et al.* 2009). One participant argued that “*The dairy breed has a lot to offer from* […an] *eating quality side of things, however it all comes down to muscle expression in those primal* [cuts]” [Interview 1]. In looking to improve the viability of surplus calves as beef animals, participants emphasised the need to select the correct beef sires. As stated by one participant: “*If they’re using some good genetics in that beef side, their* [offspring] *is going to be a lot more marketable and a lot more favourable*” [Interview 1]. The use of sexed semen in combination with beef crossbreeding has been promoted as a ‘responsible’ breeding strategy (AHDB 2020).

In discussing the marketing of dairy beef animals, participants struggled with the merit of branding products as beef that originates from the dairy herd. As one participant stated:

“*I know there’s been talk about coming out with a dairy beef brand and* [trying to] *market* [it]. *I don’t think you want to highlight the fact that it comes from dairy […] if you don’t have to tell* [consumers]*, why tell them? I think* [if you do] *you’d be giving yourself a discount on the beef price before you start*” [Interview 3].

Along the same line, some conveyed that “*the* [dairy] *producers […] want to get paid a premium for it because they think they’ve got a premium product. Well at the moment they haven’t*” [Interview 4]. Generally, participants emphasised that achieving sustainable markets was dependent on *“… going out and doing the homework around your markets and finding out if there is [1] a customer, a retailer or a consumer that is going to take the product you’ve got, to get it through the door,* [and] *[2] whether they’re going to pay you a premium for it*” [Interview 4]. Whilst beef from dairy breed animals tends to rate highly in terms of meat quality, red meat yield has been a primary reason for packer discounts on dairy beef. Although evidence of dairy beef being marketed at consistent premium with scale is lacking, it has been shown that beef produced from surplus dairy calves can be successfully marketed alongside conventional beef products when strategically bred and fed for pre-determined markets (Foraker *et al.* 2022).

Given that beef from the dairy herd already makes up a large percentage of global supply (Greenwood 2021; Foraker *et al.* 2022) where it is largely not distinguished from beef originating from beef herds, it is unsurprising that value chain stakeholders expressed doubts over the ability to create ‘new’ markets for dairy beef in Australia. Indeed, marketing beef as dairy origin may pose additional risks, should citizens become aware that differences exist in terms of whether the calf was reared by its mother, as is the routine in the beef industry, or immediately separated, as is routine in the dairy industry (Ventura *et al.* 2016).

#### Logistical and practical challenges of alternatives to early life killing

When discussing the implementation of alternatives to early life killing, participants highlighted several challenges involved with rearing the calf from birth to finishing. It has been well documented by others that the rearing of surplus calves is fraught with health and welfare challenges (for a review, see Creutzinger *et al.* 2021). Participants also noted that, to date, Australia does not have a well-established dairy beef calf rearing industry and that “*it’s the next step in the chain that needs to be built”* [Interview 6]. The lack of rearing capacity is likely a consequence of the perceived economic risk associated with rearing surplus calves for beef (Vicic *et al.* 2022). Participants also acknowledged that long-term success would require having different options for different farm businesses, recognising that *“Some people are going to be happy to rear the calves* [while others] *want to send them to a calf-rearer”* [Interview 6].

Inadequate calf-rearing capacity and knowledge were viewed as key challenges in implementing sustainable alternatives to early life slaughter. Artificial rearing of dairy calves in the absence of the dam requires careful attention to health, environment, nutrition and welfare (Mee 2008). Indeed, challenges already exist in the care of replacement heifers; for example, 38% of Australian calves failed to receive adequate transfer of passive immunity (Vogels *et al.* 2013) despite concerted industry extension efforts to improve colostrum management. Evidence exists that surplus calves are less likely to receive appropriate colostrum management compared to replacement heifer calves (Shivley *et al.* 2019). Additional challenges highlighted by participants included “*biosecurity and* [… in some cases] *20% mortality rates and probably 50% morbidity rates so half of all the calves that arrive on the farm have to be treated for some condition and 20% of them are dead*” [Interview 5]. Indeed, managing biosecurity and other stressors, such as mixing and transport, particularly when sourcing calves from multiple birth farms to rear in a central location are well known contributors to increased risk of morbidity and mortality (Renaud *et al.* 2018; Wilson *et al.* 2020).

Participants also noted that highly seasonal calving systems may overwhelm either calf rearing capacity on-farm, or an abattoir’s capacity for bobby calf slaughter, collectively increasing at-birth euthanasia. For example, “*One of the issues in Tasmania has been that there is only really one place that they process dairy calves and they are massively into a spring and an autumn* [calving] *drop* [in that region]*. […] so the abattoir cannot handle them all and they just have to get knocked on the head”* [Interview 3]. Unsurprisingly, regions in Australia with more seasonal calving and more Jersey and Holstein-Jersey cross cows have higher rates of early life killing of surplus calves and less dairy beef production (Dairy Australia 2023a).

Participants also commented on the volatility of the Australian climate (Raedts *et al.* 2017) and that *“there is always competing priorities, if there’s not a drought it’s a flood and if it’s not that it’s the price of produce and what’s happening in other countries and exports and things like that”* [Interview 7]. The impact of seasonal variation and other market factors on commodity price volatility has been reported as a major contributor to the economic viability of the industries dependent on surplus calves (Raedts *et al.* 2017). The impact of price volatility on confidence in the long-term economic viability of dairy beef value chains was made clear by one participant:

*“Like every time when we go through those big spikes in beef prices, everyone goes ‘oh I can buy a calf at 20 kgs for 700 bucks’ and then by the end of the time when it comes round to selling the animal it’s only worth $1,000 in the market because it’s a dairy cross and* [they realise] *‘I’ve run that animal for 2½ years for 200 bucks’. So that’s not what we want to do”* [Interview 3].

Beef commodity prices are often lower during times of drought in Australia (Countryman *et al.* 2016), and the annual number of bobby calves slaughtered tends to be higher during low beef commodity prices (Dairy Australia 2023b). With the impact of climate change likely increasing the severity of future droughts (Vicente-Serrano *et al.* 2020), implementing long-term, sustainable dairy beef production systems will require solutions that can withstand future volatility in seasonal conditions and commodity price environments.

### Moving towards solutions

Despite the associated challenges, participants emphasised the importance of implementing viable alternatives to early life killing of surplus dairy calves. Different approaches to affecting change were explored from both an industry and farmer perspective, along with the importance of leadership and collaboration. Finally, several unique opportunities were highlighted as downstream benefits of long-term, widespread change.

#### Affecting practice change

In discussing the need to implement widespread change in a reasonable time-frame, participants acknowledged that:

*“Like in any community* [or] *industry you’ve got a percentage of people who are go-getters, change-makers, and can see the role of the industry and the benefit of it and then you’ve got a group of people in the middle* [who] *will go one way or the other depending on the difficulty or the ease and then you’ve got that bottom group of people who for whatever reason sit on their hands”* [Interview 3].

The impact of tradition and culture in the agricultural sector on farmers’ willingness to change was also highlighted. *“It’s different for different people but doing what you’ve always done and what your father or your mother did, it is a very large part of it…”* [Interview 2]. That farmers vary in their attitudes to implementing change has been recognised (Munoz *et al.* 2019), whilst the influence of tradition and self-identity on Australian producer attitudes to sustainability and adapting to change has been explored by others (Lankester 2012).

In affecting change, some participants emphasised the need to ensure ownership and buy-in beginning at the dairy farm and avoiding mandates that may incite push-back. As one participant stated *“[…] as soon as we get to the position where we’re telling people how to run their farm, they’ll tell us to bugger off and we’ll lose that connection*” [Interview 5]. However, others felt legislation may be the only way to shift the behaviours of some segments of the industry. “[There is a] *particular bottom group of people that just want to do what they want to do, how they want to do it, because that’s how they’ve done it. Something like legislation would impact them*” [Interview 3].

Participants also discussed the efficacy of legislation and policy, using blunt force trauma euthanasia as an example, a practice that has been prohibited in Australian industry policies (Australian Dairy Farmers 2020) and legislation (Animal Health Australia 2016).

“*I am concerned by the data on blunt-force trauma* [euthanasia] *because we have a […] very clear policy position, yet our survey data doesn’t say we’re complying with* [it…]. *That always concerns me about what is the true appetite to take what you’re going to tell us and push it into a sufficient level of implementation to be able to further mitigate the risks around bobby calves or calf management*” [Interview 2].

#### Role of leadership and collaboration

When contemplating sustainable change, participants highlighted the need for industry-level leadership to ensure that sector-wide change is achieved. Desire to take leadership on the issue from singular stakeholders, whilst important, will alone be unlikely to address the problem. Despite initial efforts by dairy farmers in Atlantic Canada to develop a viable dairy beef industry, numerous challenges were identified indicating the complexity of the undertaking (Proudfoot *et al.* 2022). Our participants stated that any leadership initiatives should be *“[…] concrete* [with] *a clear commitment”* [Interview 2].

The role of an industry-wide commitment to ending early life slaughter was seen as valuable in underpinning community trust. Some viewed an industry-wide commitment as being an integral step in uniting the industry and allowing stakeholders to hold each other accountable; thereby protecting, at least in part, the dairy industry’s social licence to operate.

Though not a new concept, social licence to operate, has grown in popularity in recent years with some arguing it is just another term for legitimacy (Gehman *et al.* 2017). For alternatives to early life killing of surplus calves to be perceived as legitimate, all stakeholders, including citizens, farmers and the wider dairy industry, must all view solutions as acceptable and viable (Gehman *et al.* 2017). The notion that industry (and not government) should lead the process of change was clearly stated by one participant who desired “*An industry goal* [where each member of the value chain has] *a little bit of skin in this game* [ensuring that we do] *the right thing…”* [Interview 6].

#### Downstream benefits of dairy beef production

Participants noted unique opportunities in implementing dairy beef production systems as alternatives to early life killing of surplus calves. Amongst these were the ability for increased production of beef from the dairy herd to improve business resilience. One participant stated that: *“I think […] the really nice thing about where we are now is sustainability, people love the circular economy, they want the whole thing end to end and improving supply chains and improving your* [carbon] *footprint”* [Interview 7]. Similarly, Romera *et al.* (2020) highlighted the opportunities in redesigning dairy production systems as a co-ordinated food production network instead of continuing to operate as an isolated entity. The concept of circular bioeconomy business models has also been found to offer opportunities as well as challenges (D’Amato *et al.* 2020).

Participants also highlighted the potential advantages to be gained from producing dairy beef in reducing carbon emissions (Tichenor *et al.* 2017; van Selm *et al.* 2021). The International Dairy Federation’s prescribed carbon accounting methodology stipulates that 85% of a dairy cow’s annual emissions are attributable to her milk, leaving just 15% attributable to her dairy beef calf (IDF 2022); this provides a potential advantage to dairy beef value chains over beef originating from the beef herd. In the words of one participant:

*“We need to ultimately try and decide what’s the best solution that’s going to* [find] *fitness for purpose in every animal that we produce and from our whole of food production sustainability perspective we’ve got these cows producing milk. If a high proportion of them are also producing beef from a calf, we’re reducing our environmental footprint collectively as well”* [Interview 3].

### Study limitations and future research

Whilst the present study included perspectives from a range of stakeholders across the Australian beef and dairy value chains, a limitation of the research is that each stakeholder group was only represented by one, or in some cases two, organisations. Thus, our findings are not intended to be generalisable across the Australian dairy or beef value chains, nor the global network of beef and dairy industry stakeholders. Despite this, the inductive qualitative methodology used (Braun & Clarke 2006) allows for an understanding of the complex frame of reference through which our participants understood the surplus calf challenge. We encourage further participatory work that includes the voices of all stakeholders, including industry, the public, and the animals, to enable improved understanding of the surplus dairy calf challenge.

## Animal welfare implications and conclusion

Understanding the attitudes of beef and dairy industry stakeholders to surplus dairy calf management in Australia is critical to implementing socially and economically sustainable alternatives to early life killing. Participants in this study identified that animal welfare outcomes can be both positive or negative whether surplus calves are killed in the first few days of life or raised for beef, indicating that the challenge is complex. However, finding sustainable ways to shift the perception of a class of production animal away from a being waste product and into being seen as a valued commodity is likely to have widespread benefits to the standard of care received by the animals, thereby improving welfare standards.

## Supporting information

Bolton et al. supplementary material 1 Bolton et al. supplementary material

Bolton et al. supplementary material 2 Bolton et al. supplementary material

## Supplementary material

The supplementary material for this article can be found at http://doi.org/10.1017/awf.2024.4.
