# Supplementary material for: Waste not, want not: Value chain stakeholder attitudes to surplus dairy calf management in Australia
Source: Anim Welf. 2024 Feb 22;33:e10. doi: 10.1017/awf.2024.4 (PMC10951662; doi:10.1017/awf.2024.4)
Supplement: Bolton et al. supplementary material 1 — Bolton et al. supplementary material [file S0962728624000046sup001.pdf]

Codebook

Theme	Subtheme	Subtheme description
Ethics of surplus calf management	Concerns about societal views	Perception of calf surplus as a problem because of public ethical concern for animal welfare.
	Personal views of industry stakeholders	Arguments regarding the impact of surplus calf management practices on the personal lives of farmers and supply chain stakeholders.
	Animal welfare versus ethics and public perception	Views on the ethics of different practices (e.g. early life slaughter vs on-farm euthanasia) and their impact on animal welfare and public perception.
Economics of surplus calf management	Disunity amongst stakeholders	Perception of divergent views from different stakeholder groups about surplus calves.
	Quality and productivity of dairy beef	Reasons for the reduced product quality of dairy beef calves and surplus dairy calves.
	Logistical and practical challenges of alternatives to early life killing	Pre-farm, on-farm and post-farm challenges faced by farmers and other stakeholders when rearing, managing and marketing surplus calves.
Moving towards solutions	Affecting practice change	Attitudes of supply chain stakeholders towards creating change to achieve a solution to the surplus calf issue.
	Role of leadership and collaboration	Positive economic, environmental and practical outcomes of achieving a sustainable solution to the surplus calf issue.
	Downstream benefits of dairy beef production	Views about the importance of leadership and initiative roles, as well as collaborative commitment of all stakeholders.
