# Supplementary material for: Waste not, want not: Value chain stakeholder attitudes to surplus dairy calf management in Australia
Source: Anim Welf. 2024 Feb 22;33:e10. doi: 10.1017/awf.2024.4 (PMC10951662; doi:10.1017/awf.2024.4)
Supplement: Bolton et al. supplementary material 2 — Bolton et al. supplementary material [file S0962728624000046sup002.pdf]

Interview guide

Opening
Before we get going it would be great to get an idea of your understanding of the bobby calf issue?
Has your organisation ever investigated how to address this issue?
Project Overview
SB provides a 10-minute overview of the project and where we are up to:<br>*This project aims to work with industry stakeholders and members of the community to implement alternatives to early life slaughter of surplus calves (bobby calves) that are not just financially viable, but socially acceptable.*<br>*The management of surplus calves is complex, with many opportunities but many persistent challenges. This project will pilot a new approach to developing alternatives that recognises that the people who face a problem every day are the ones that hold the key to sustainable solutions. These solutions may have a direct benefit for farm businesses as well as the wider Australian dairy industry through farm output diversification, potentially increased revenue, improved market access and improved community trust.*<br>*This project will be conducted in two phases. In Phase 1 of the project, an 'industry' group will take part in several facilitated discussions designed to explore attitudes to surplus calf management, perceived factors contributing to the status quo, barriers to practice change and possible methods for overcoming these barriers. At the same time, the project will hold separate 'community' group discussions, designed to explore the attitudes of the general public to how we manage calves now, and what will be socially acceptable in the future. Interviews will also be held with supply chain stakeholders, in which you are invited to participate today.*<br>*Through professional facilitation, the industry group will use findings from the community group workshops, supply chain stakeholder interviews, as well as their own discussions and learnings, to identify barriers to implementing sustainable surplus calf management. Phase 2 of the project provides funding for a secondary piece of work to address the rate limiting barrier/s identified in Phase 1.*
Feedback
Interested in your high-level feedback on the approach outlined?
We are interested in your top line thoughts on the four pathways that we tested with consumers. What would work/ wouldn't with your customers? Just a reminder these aren't the pathways developed out of the program, rather these are ideas commonly suggested. We will be designing potential pathways for the South coast dairy region in our June workhsop.
1. All surplus calves are grown to adulthood for the beef supply chain. This is able to be done because the costs are covered either through milk price increase or supply chain co-operation.

2. Hormones are introduced to enable cows to continue to produce milk without having to repeatedly become pregnant, meaning surplus calves are never born in first place.
3. The South Coast dairy industry transforms into a connected network of farms that work together on a solution. The solution differentiates the region’s milk and/or dairy beef and attracts a premium.
4. Farmers use technology that is currently available including sexed semen to reduce male calves being born. The remaining calves either enter the dairy industry or are grown to adult hood with optimised nutrition and production for the beef supply chain.
Role in issue
From your perspective is there a part of the supply chain that should be responsible for addressing this issue?
Is cooperation and collaboration along the supply chain required?
Is supply chain cooperation on this issue something that your organisation would be interested in exploring?
Would this be limited to the South Coast or are there other dairying regions you'd be interested in being involved in exploring solutions?
Do you think that solutions will differ between regions?
Other Comments
Any other comments?
Would you like to stay informed/ engaged/ other?
